# Post-stenotic aortic dilatation

**DOI:** 10.1186/1749-8090-1-7

**Published:** 2006-03-03

**Authors:** Emma Wilton, Marjan Jahangiri

**Affiliations:** 1 Department of Cardiothoracic Surgery, St. George's Hospital and Medical School, London, UK

## Abstract

Aortic stenosis is the most common valvular heart disease affecting up to 4% of the elderly population. It can be associated with dilatation of the ascending aorta and subsequent dissection. Post-stenotic dilatation is seen in patients with AS and/or aortic regurgitation, patients with a haemodynamically normal bicuspid aortic valve and following aortic valve replacement. Controversy exists as to whether to replace the aortic root and ascending aorta at the time of aortic valve replacement, an operation that potentially carries a higher morbidity and mortality.

The aetiology of post-stenotic aortic dilatation remains controversial. It may be due to haemodynamic factors caused by a stenotic valve, involving high velocity and turbulent flow downstream of the stenosis, or due to intrinsic pathology of the aortic wall. This may involve an abnormality in the process of extracellular matrix remodelling in the aortic wall including inadequate synthesis, degradation and transport of extracellular matrix proteins.

This article reviews the aetiology, pathology and management of patients with post-stenotic aortic dilatation.

## Methods

An English literature search using Pubmed-Medline database between 1960 and today was carried out. Key words used included aortic valve, aortic stenosis, aortic dilatation, bicuspid aortic valve, surgery and matrix metalloproteinase.

### Definition

Aortic stenosis (AS) is the most common valvular heart disease affecting up to 4% of the elderly population [1,2]. Post-stenotic aortic dilatation is defined as dilatation of the vessel wall distal to the area of a partial stenosis. It refers to dilatation of the ascending aorta, >4.0 cm, distal to a stenotic/malformed aortic valve (AV). This dilatation is usually progressive > 0.3 cm/year.

Aortic dilatation is thought to be a precursor to aortic dissection and rupture, both of which are potentially fatal.

### Aetiology

Post-stenotic aortic dilatation has been shown to occur in patients with AS/aortic regurgitation (AR), haemodynamically normal bicuspid aortic valve (BAV) and following aortic valve replacement (AVR). It does not appear to be related to the degree of AS [3], although this study was conducted on patients with a valve area < 2.0 cm^2^, and appears to be independent of whether the patient has had valve replacement [4]. This suggests a possible genetic basis for the dilatation as well as the mechanical stresses placed on the vessel wall downstream of a stenotic lesion.

BAV is an independent risk factor for both AS and progressive aortic dilatation [5].

### Aortic stenosis (AS)

Aortic stenosis is the most common valvular heart disease and the third most common heart disease, after hypertension and coronary artery disease, in Europe and North America. In the elderly the prevalence of aortic stenosis has been reported to be up to 4%. Aortic sclerosis, the precursor of aortic stenosis, has been found in approximately a third of patients over the age of 65 years [1,2]. In most patients the underlying cause is calcific AS [6]. This is a chronic progressive disease that begins with thickening and calcification of the valve cusps without haemodynamic significance and ends in heavily calcified, stiff cusps that cause severe valve stenosis. Recent studies have shown that this is not only a degenerative process due to mechanical stress, but also an active process, involving inflammation and lipid infiltration, similar to that seen in atherosclerosis. Epidemiological studies have confirmed that AS and atherosclerosis share several common risk factors: male sex, older age, hypertension, diabetes, smoking and elevated levels of low-density lipoprotein (LDL) cholesterol and lipoprotein(a) [1,7]. These observations have led to the proposal of pharmacological strategies, already used in atherosclerosis, e.g. angiotensin-converting enzyme inhibitors and hydroxymethylglutaryl-coenzyme A reductase inhibitors (statins), which may slow the progression of AS. However, the SALTIRE study [8] has shown results contrary to this. This was a double-blind, placebo-control trial (n = 155) in which patients were treated with 80 mg of atorvastatin or matched placebo. They were assessed at follow up (median 25 months) for AS and AV calcification by echocardiography and serum LDL cholesterol. They concluded that intensive lipid-lowering therapy did not halt the progression of calcific aortic stenosis or induce its regression but that large, long-term, randomised trials were needed.

### Bicuspid aortic valve (BAV)

BAV is a common congenital abnormality found in adults. It occurs in approximately 1–2% of the population [9,10]. This compares to 0.8% of all other forms of congenital cardiac disease combined. The frequency of BAV is higher in males (male: female ratio, 2:1). Approximately a third of BAV patients develop serious complications. Therefore, it causes more morbidity and mortality than the combined effects of all other cardiac conditions [9,11]. BAV is associated with premature valve stenosis, regurgitation, infective endocarditis, ascending aortic aneurysms and dissection. Nearly all patients with a BAV will require valve surgery during their lifetime and it has been suggested that an underlying congenitally malformed valve is more common than a tricuspid aortic valve (TAV) as the underlying cause for isolated AVR for AS in adults [12].

## Pathogenesis of BAV

### Embryology

BAV is the result of abnormal aortic cusp formation in valvulogenesis. There is fusion of adjacent cusps to form a single aberrant cusp, which is larger than the one remaining normal-sized cusp but smaller than 2 normal cusps combined. It is therefore likely that BAV is the result of a complex developmental process and not simply the fusion of 2 normal cusps. The larger leaflet has a false commisure that, on histological examination, shows no valve tissue. It is thought that congenital AV malformations maybe a phenotypic continuum of unicuspid (severe form), bicuspid (moderate form), tricuspid (normal) and the rare quadricuspid forms [13,14].

BAV is associated with coarctation of the aorta, patent ductus arteriosus and left main stem stenosis which supports a genetic cause for the disease [15,16]. There is also a high incidence of familial clustering of BAV, compatible with autosomal dominant inheritance with reduced penetrance [17].

### Flow related theory

This theory sites abnormal blood flow through the AV during valvulogenesis resulting in abnormal cusp separation and the formation of a BAV. There is, however, no concrete evidence to support this theory.

### Support for intrinsic aortic disease leading to post-stenotic dilatation

Patients with BAV have a larger ascending aortic diameter compared to age and sex-matched control subjects, irrespective of altered haemodynamics [18]. The left ventricular outflow tract, aortic cusps, arterial media of the ascending aorta and aortic arch are all linked embryologically as they all originate from the neural crest [19]. Disorders of the neural crest have also been implicated in the development of cervicocephalic arterial dissection. A familial cluster of aorto-cevicocephalic arterial dissection and BAV has been described strengthening the theory of an underlying neural crest defect in the development of BAV [20].

Endothelium-derived nitric oxide (NO) plays a role in valvulogenesis as well as cell growth and apoptosis, post developmental vascular remodelling and angiogenesis. Mice deficient in endothelial NO synthase, which synthesizes endothelium-derived NO, were found to have a significantly high incidence of BAV [21]. This suggests that the genetic determinants of BAV are linked to the genetic determinants of arterial abnormalities.

NO has also been implicated in regulating the expression of matrix metalloproteinase-9 in the aortic wall of rats. It may, therefore, be involved in the homeostasis between matrix metalloproteinases (MMPs) and tissue inhibitors of metalloproteinases (TIMPs); a deficiency of NO tending towards matrix degradation [22].

Gurvitz et al [23] studied 2 groups of patients, one with isolated BAV (n = 76) and the other normal tricuspid AV (TAV) (n = 41), under the age of 21 years old, diagnosed using transthoracic echocardiography. Patients were excluded if they had other cardiac anomalies, a diagnosis of Turner's or Marfan's syndrome, or a surgical or catheter-based aortic valve intervention. Aortic root dimensions at the annulus, sinus of Valsalva, sinotubular junction (STJ) and the proximal ascending aorta were assessed in the parasternal long-axis view in systole. The haemodynamic state of the AV was evaluated using colour flow and spectral Doppler. In normal subjects it was seen that aortic root dimensions correlated well with height and body surface area (BSA), better than with age. It was also seen that at every level of the aortic root, in patients with isolated BAV, independent of the functional state of the valve, the diameter was significantly greater than normal TAV, but within the normal range (Table 1).

**Table 1:** Mean z scores, in relation to height, in children with BAV compared to those with TAV.

**Study**	**N**		**Ao Ann**		**Ao Sinus**		**STJ**		**Asc Ao**	
	**BAV**	**TAV**	**BAV**	**TAV**	**BAV**	**TAV**	**BAV**	**TAV**	**BAV**	**TAV**

**Gurvitz ^23^**	76	41	2.0	0	1.6	0	1.2	0	3.3	0
	**p Value**		< 0.001		< 0.001		< 0.001		< 0.001	

Ao Ann = aortic annulus, Ao Sinus = aortic sinus, STJ = sinotubular junction, Asc Ao = ascending aorta.

BAV = Bicuspid aortic valve, TAV = Tricuspid aortic valve

Mean circumferential stress in the dilated ascending aorta increases linearly with blood pressure and diameter. Distensibilty has been shown to be due mainly to the intrinsic elastic properties of the aorta itself. Different groups of patients studied, including patients with Marfan's syndrome and BAV with associated aneurysm, have different predicted distensibility. In patients with BAV, it was seen that valve function did not influence either the elastic properties or the distensibility of the aorta suggesting again that it is the intrinsic abnormalities within the wall of the aorta, and not abnormal flow patterns, which lead to aortic dilatation [24].

### Aortic dilatation associated with haemodynamically normal AV

Nkomo et al [25] carried out a community-based study to determine whether the association between BAV and aortic dilatation could be demonstrated in patients with BAV, without significant stenosis or regurgitation. Patients were identified by echocardiography. They were excluded if there was evidence of AS, more than trivial AR, aortic coarctation, or mitral, pulmonic or tricuspid valve disease, cardiomyopathy, pericardial disease, Marfan's syndrome or a family history of Marfan's syndrome, or any other form of congenital heart disease. 44 patients were matched to an equal number of controls with normal TAV of the same age (mean 35 ± 13 years), sex (65% male) and BSA. Aortic dimensions were measured at the annulus, aortic sinus, proximal ascending aorta and aortic arch. It was found that the dimensions of the aortic root were consistently larger in patients with BAV. The largest difference was seen in the dimensions of the proximal ascending aorta. There was no significant difference between the BAV and control groups with respect to dimensions of the aortic arch. Other studies have confirmed these findings, of dilatation of the aortic root and ascending aorta in patients with BAV, and have also shown that there is no increased dilatation seen in the descending or abdominal aorta [26,27] (Table 2).

**Table 2:** Aortic dimensions (mm) in patients with haemodynamically normal AV.

**Study**	**N**		**Ao Ann**		**Ao Sinus**		**Asc Ao**		**Ao Arch**	
	**BAV**	**TAV**	**BAV**	**TAV**	**BAV**	**TAV**	**BAV**	**TAV**	**BAV**	**TAV**
**Nkomo ^25^**	44	44	23.2	21.6	33.5	30.3	33.3	27.9	24.2	25.3
			p = 0.002		p = 0.0001		p = 0.0001		p = 0.16 (N/S)	
**Nistri ^26^**	66	70	23.6	22.7	31.6	28.7	31.2	26.9	N/A	N/A
			p = N/S		p < 0.001		p < 0.001			
Cecconi ^27*^	162	162	15.4	14.4	22	19.5	23.7	18.6	15.1	14.4
			p = N/S		p < 0.01		p < 0.001		p = N/S	

* Dimensions in mm/m^2^

Ao Ann = aortic annulus, Ao Sinus = aortic sinus, Asc Ao = ascending aorta, Ao Arch = aortic arch.

BAV = Bicuspid aortic valve, TAV = Tricuspid aortic valve

N/A = not applicable, N/S = not significant

### Aortic dilatation associated with aortic stenosis

Crawford and Roldan [3] carried out a study to determine the prevalence of dilated aortic root in patients with AS. They studied the echocardiograms of 118 patients with AS, with a valve area of <2.0 cm^2^. They were age-matched to patients with aortic sclerosis, but no stenosis, and to normal controls. The aortic root diameter at the annulus, coronary sinus and STJ levels were measured using transthoracic echocardiography. Dilated aortas were defined as 2 SDs above the mean values obtained in the normal group. This study concluded that aortic root dilatation is common in AS, but is not related to severity of the stenosis. They did not, however, make any comparison between BAV and TAV. As stated earlier, one of the limitations of this study is that only patients with a valve area < 2.0 cm^2 ^were included in the study.

Morgan-Hughes et al [28] carried out a prospective study measuring the aortic root and ascending aortic diameters, using CT scan images, on patients (BAV, n = 10; TAV, n = 18) with severe AS prior to undergoing AVR. They concluded that patients with BAV and pure, severe AS have moderately dilated thoracic aorta compared to matched TAV (p < 0.005) (Table 3).

**Table 3:** Aortic dimensions (mm) in patients with AS

**Study**		**N**		**Ao Ann**		**Ao Sinus**		**STJ**	
**Crawford ^3^**	**No AS**	108		25		36		30	
	**Mild AS**	47		25		36		36*	
	**Moderate AS**	29		24		36		36*	
	**Severe AS**	42		24		36		35*	
		**BAV**	**TAV**	**BAV**	**TAV**	**BAV**	**TAV**	**BAV**	**TAV**
**Morgan-Hughes ^28^**		10	18	N/A	N/A	41.1	33.8	39.1	31.1
	**p Value**					< 0.005		< 0.005	

*p < 0.001 compared to normal/sclerotic AV.

Ao Ann = aortic annulus, Ao Sinus = aortic sinus, STJ = sinotubular junction.

BAV = Bicuspid aortic valve, TAV = Tricuspid aortic valve

N/A = not applicable.

Keane et al [29] carried out a retrospective study to compare aortic size in BAV and controls with matched valvular lesions (AS, AR or mixed lesions). They measured the diameter of the left ventricular outflow tract (LVOT), sinus of Valsalva, STJ and proximal ascending aorta using transthoracic echocardiograms. 118 consecutive patients with BAV were compared to controls with TAV. Paired analysis demonstrated significant aortic dilatation at all levels measured in BAV patients, with all degrees of AS and/or AR, compared to controls (Table 4). These differences were seen despite the significantly older age of the control group compared to the BAV group (mean 55.3 years (TAV) and 43.9 years (BAV)). This study also confirms the result mentioned above, that the degree of dilatation is not related to the severity of AS. It suggests that post-stenotic dilatation is a feature of BAV but not of congenitally normal valves. It supports the theory that this is due, in part, to an underlying pathology within the aortic wall.

**Table 4:** Aortic dimensions (mm) of matched patients with AS/AR/Mixed valve disease.

**Study**	**N**		**Ao Sinus**		**STJ**		**Proximal Aorta**	
	**BAV**	**TAV**	**BAV**	**TAV**	**BAV**	**TAV**	**BAV**	**TAV**
**Keane ^29^**	77	77	37.4	35.1	35.4	33.4	36.1	34.0
	**p Value**		< 0.01		< 0.001		< 0.001	

Ao Sinus = aortic sinus, STJ = sinotubular junction.

BAV = Bicuspid aortic valve, TAV = Tricuspid aortic valve

### Aortic dilatation occurring post AVR

Patients that undergo AVR often have some degree of ascending aortic dilatation. At present, replacing the aortic root at the time of AVR is controversial as the risk of aortic dilatation following AVR is uncertain. Some studies have been carried out to determine the natural history of ascending aortic dilatation following AVR.

Yasuda et al [4] carried out a retrospective analysis of patients (BAV n = 13, TAV n = 14), using echocardiography, before and after AVR and 18 BAV patients without AVR. Diameters were measured at the sinus of Valsalva, STJ and the proximal ascending aorta. The annual dilatation rate was calculated by dividing changes of diameter during the follow-up period by the BSA and the observational interval. Aortic dilatation in BAV patients was seen to be significantly faster than that of TAV at the proximal aorta only (0.18 compared to -0.08 mm/m^2^/year). There was no significant difference in the dilatation rates of BAV patients with and without AVR (0.03 and 0.02 mm/m^2^/year respectively). This study showed that AVR could not prevent progressive aortic dilatation in BAV. However, TAV patients did not show further aortic dilatation after AVR. It therefore seems that haemodynamic factors are not responsible for the ongoing dilatation in BAV.

### Genetic/structural abnormality

Associations have been found between post-stenotic dilation and genetic and structural abnormalities within the AV and ascending aorta, as set out below. However, there is a lack of randomised trial data to establish whether these associations are in fact causal.

### Fibrillin-1

Fibrillin-1 is a major protein component of extracellular matrix fibrils, called microfibrils. The FBN1 gene, found on chromosome 15, encodes for this large glycoprotein. Vascular tissues with deficient fibrillin-1 microfibrils release metalloproteinases. These enzymes weaken the vessel wall by degrading the elastic matrix components and leading to matrix disruption and consequent dilatation of the vessel.

Marfan's syndrome results from mutations in the FBN1 gene. The most common cardiovascular complication of this condition is progressive aortic root enlargement, initially occurring at the sinus of Valsalva, and ascending aortic aneurysms. The aneurysms develop as a consequence of disruption of the medial and adventitial elastin and collagen in association with focci of cystic medial necrosis of the medial smooth muscle. There is also seen to be increased expression of MMPs (especially 2 and 9) within the aneurysmal aorta and, to a lesser extent, the AV [30].

Fedak et al [31] carried out a study to assess the vascular matrix remodelling in patients with BAV and its implication for aortic dilatation. Samples of aorta and pulmonary artery were obtained from patients undergoing surgery with BAV (n = 21) and TAV (n = 16). The amount of fibrillin-1, elastin and collagen was determined using quantitative immunohistochemical analysis, using fluorescence microscopy, for fibrillin-1, and hydroxyproline determination. Fibrillin-1 content was significantly reduced, in both the aorta and pulmonary artery, of BAV patients compared to that seen in TAV suggesting a systemic deficiency of this. It was independent of valve function and patient age. However, the amount of matrix components, elastin and collagen, were unchanged. This decrease in the amount of fibrillin-1 in the vasculature of patients with BAV may trigger MMP production leading to matrix disruption and vascular dilatation.

Recent studies have demonstrated that there is a difference in the expression of matrix proteins between the convexity and concavity of the dilated aorta in patients with BAV [32]. These results are consistent with the wall-stress asymmetry that has also been reported by Robicsek and colleagues [33]. They studied three explanted haemodynamically normal congenitally BAV, where they used a simulator to produce a computerised digital model. They showed excessive folding and creasing persistent throughout the cardiac cycle, extended area of leaflet contact, significant morphologic stenosis and asymmetrical flow patterns and turbulence. Furthermore, Richards et al [34] studied the influence of structural geometry on the severity of BAV and stenosis using transthoracic, transoesophageal echocardiography and computer simulations. They demonstrated that for the same anatomic orifice area, functional severity is greater in BAV than in degenerative TAV patients with AS.

### Matrix metalloproteinases (MMPs)

The MMPs are a family of proteases that play an important role in the homeostasis of connective tissue. They are synthesized by a variety of cell types including endothelial and smooth muscle cells, fibroblasts and macrophages. They are classified into classic MMPs and novel MMPs. The classic MMPs are secreted as proenzymes and include 3 subgroups: (1) the interstitial collagenases (MMP-1, MMP-8 and MMP-13), which degrade fibrillar collagen; (2) the gelatinases (MMP-2 and MMP-9), which act on type IV collagen and partially degrade fibrillar collagens as well as elastin; and (3) stromelysins (MMP-3, MMP-7 and MMP-10), which have a broad substrate specificity. A metalloelastase (MMP-12), which degrades elastin, is also included in this group. The novel MMPs are secreted in active form or are associated with the cell surface. The activity of the MMPs is regulated by their interaction with 4 types of tissue inhibitors of metalloproteinases (TIMPs). The balance between MMPs and TIMPs regulates the degradation of extracellular matrix in normal and pathological states [35-37]. TIMP-1 is the most common in the aorta. It is a 25-kd polypeptide capable of inhibiting most MMPs and is produced by fibroblasts or smooth muscle cells.

Degradation of ECM, especially elastin, within the aortic wall is a hallmark of abdominal aortic aneurysms (AAA). Studies have identified increased expression of MMP-1, MMP-2, MMP-9, TIMP-1, TIMP-2 and MMP/TIMP ratios as an important factor in the aetiology of AAA formation. However, the predominant MMP expressed in AAA is MMP-9, produced by macrophages [38].

Elastin and collagen degradation in thoracic aortic aneurysms is mediated by MMPs, particularly the gelatinases, MMP-2 and MMP-9.

Le Maire et al [39] studied MMP expression in ascending aortic aneurysms associated with BAV and TAV. Samples of ascending aorta were obtained from 29 patients (BAV n = 14, TAV n = 15). Histological and immunohistochemical analysis was carried out on the specimens. They showed that ascending aortic aneurysms exhibited increased MMP expression when compared to controls (non-aneurysmal aortic tissue). The pattern of MMP expression, however, differed between aneurysms associated with BAV and those with TAV. Another study, carried out by Boyum et al [40], showed that there was an increase in the level of MMP-2 and MMP-9 in thoracic aortic aneurysms associated with BAV compared to TAV. They did not, however, find any significant difference in the expression of TIMP-1 or TIMP-2.

Most studies have looked at the increased expression of MMPs within aneurysmal tissue. Ikonomidis et al [41] studied the effects of deletion of the TIMP-1 gene on the progression of murine thoracic aortic aneurysms (TAA). They used adult wild-type and TIMP-1 knockout mice. They showed that deletion of the TIMP-1 gene resulted in an increase in size and continued progression of TAA formation compared with wild-type mice. They concluded that this was, at least in part, due to the alteration of the balance between gelatinase activity and its endogenous inhibition. These results suggest that therapeutic strategies that can shift the MMP/TIMP stoichiometric balance away from net proteolysis may be used to inhibit the incidence and progression of TAA.

In diseased or degenerated congenitally deformed valves, increased local MMP activity could alter their elastic and collagen component leading to structural and functional failure.

Histological and immunohistochemical analysis of the AV in patients with nonrheumatic TAV and AS revealed an inflammatory infiltrate within the AV leaflets and an increase in expression of MMP-1, MMP-2 and MMP-3 in AV of patients with severe AS. It was also found that MMP-9 was only present in the leaflets of patients with AS [42,43].

Koullias et al [44] carried out a study to semiquanitatively analyse the expression of MMPs (1, 2 and 9) and TIMPs (1 and 2) in AV tissue. The study group consisted of 26 patients, undergoing surgery for AS, AR, ascending aortic aneurysm or type A dissection (BAV n = 10, TAV n = 16, controls n = 4). They showed that MMP-9 expression was significantly higher in BAV compared to normal and diseased TAV. This increased proteolytic presence in BAV may lead to the observed decrease in elastin and collagen content and their resultant functional failure. Others have reported similar results [45].

In summary, these findings suggest that an increase in MMP expression in the aorta of BAV patients may explain the predilection to aneurysm formation. Therefore, by modifying the MMP activity, it maybe possible to reduce or prevent the progression of thoracic aortic aneurysms (TAA). There may also be a role for altering the expression of MMPs to help reduce the progression of BAV disease.

### Histological abnormalities

Congenital BAV is associated with cystic medial necrosis, that is, necrosis of the medial smooth muscle cells and accumulation of proteoglycan, of the aorta. De Sa et al [46] examined the histological changes in the ascending aorta and pulmonary trunk in patients with BAV. They studied 31 patients (BAV, n = 20 and TAV, n = 11) undergoing AVR. Samples of ascending aorta and pulmonary trunk were collected at the time of surgery. The degenerative changes (medionecrosis, fibrosis, cystic medial necrosis (mucoid material accumulation), changes in smooth muscle cell orientation and elastic fragmentation) in the ascending aorta and pulmonary trunk of patients with BAV disease were significantly more severe than in TAV patients. This severity was mainly related to degree of cystic medial necrosis, smooth muscle cell changes and elastic fragmentation. These findings may explain aortic root and ascending aortic dilatation in patients with BAV disease and pulmonary autograft dilation in certain patients following the Ross procedure.

### Ascending aortic dilatation post AVR

Replacement of the ascending aorta at the time of AVR is controversial because the risk of progressive dilatation following AVR is uncertain (Table 5). Andrus et al [47] set out to determine the natural history of ascending aorta dilatation following AVR. They studied 185 patients undergoing AVR. They measured the ascending aortic diameter, 2 cm above the sinotubular ridge, using transthoracic echocardiography before surgery and during the follow-up period. Progressive aortic dilatation was defined as an increase in diameter of >0.3 cm from the preoperative measurement. This was observed in only 15% of the study population. No patients with baseline aortic dilatation (3.5–5.3 cm) dilated > 5.5 cm during the follow-up period (n = 107, mean 33.6 months). They found no clinical or valvular characteristics that predicted progressive aortic dilatation. Their conclusion was therefore against routine replacement of the ascending aorta at the time of AVR.

**Table 5:** Ascending aorta dilatation rate following AVR

**Study**	**N**		**F/U (mo)**	**Expansion Rate (cm/year)**	
**Andrus ^47^**	185		30.0	-0.03*	
	107		33.6	-0.01*	
	**BAV**	**TAV**		**BAV**	**TAV**
	21	164	30.0	+0.14 **	-0.05
**Matsuyama ^48^**	35		97.2	+0.058*	
**Yasuda ^4^**	**BAV**	**TAV**		**BAV**	**TAV**
	13	14	116.4	+0.018***	-0.008
				cm/(m^2^/yr)	cm/(m^2^/yr)

*p = N/S, **p = 0.06, ***p = 0.03

BAV = Bicuspid aortic valve, TAV = Tricuspid aortic valve

F/U (mo) = follow-up (months)

Another clinical study, by Matsuyama et al [48], looked at the incidence of aortic complications in 35 patients following AVR, with a preoperative dilated ascending aorta = 4.0 cm, assessed by computed tomography or operative findings. The baseline aortic diameter in the study population ranged from 4.0–5.5 cm. The mean follow -up period was 8.1 ± 3.5 years (range: 2.3–13 years). Aortic events occurred in 5 patients (1 aortic dissection, 2 aortic rupture and 2 reoperations). These complications occurred in patients with baseline aortic diameters 4.7–5.0 cm. The authors concluded that the clinical course of patients with dilated ascending aorta is unpredictable and may occur even in patients with a baseline aortic diameter < 5.0 cm. They concluded that preventative aortic surgery at the time of AVR should be considered, to prevent aortic rupture and dissection, in patients with ascending aorta of 4.0–5.0 cm.

As mentioned earlier, Yasuda et al [4] showed that AVR in patients with BAV did not prevent progressive dilatation of the ascending aorta.

### Surgical implications

At present there is controversy over the best management for mild to moderate aortic dilatation associated with AV disease, especially BAV. This is partly due to the lack of concrete evidence for progressive dilatation following AVR and partly due to the increased risk associated with aortic root replacement (ARR).

A retrospective study carried out by Shapira et al [49] over a 10 year period (1987–1997) showed that advances in non-invasive diagnosis and improved perioperative management has lead to a decrease in morbidity and mortality of patients undergoing surgery on the proximal aorta with the operative mortality of thoracic aortic aneurysm repair reduced from between 9–17% to 2.6%.

### Should the ascending aorta be replaced in patients with BAV?

AVR is the treatment of choice for patients with symptomatic AV disease. Current recommendations for the surgical replacement of ascending aortic aneurysms are diameter >5.5 cm or = 5.0 cm in patients with Marfan's syndrome [50]. Borger et al [51] studied 201 patients with BAV (mean age 56 ± 15 years) who underwent AVR. Patients were excluded if they had concomitant ascending aorta replacement. All BAV with ascending aorta > 5.0 cm had ascending aortic replacement and were therefore excluded. During the follow-up period of 10.3 years, 22 patients had long-term complications related to the ascending aorta including replacement of ascending aorta for aneurysm (n = 18), dissection (n = 1) and sudden cardiac death (n = 3). 44 patients had reoperations, mainly for AV prosthesis failure. The 15-year freedom from ascending aorta-related complications was 86%, 81% and 43% in patients with an aortic diameter of <4.0 cm, 4.0–4.4 cm and 4.5–4.9 cm respectively. The authors therefore concluded that patients undergoing AVR for BAV disease should have their ascending aorta replaced if the preoperative diameter is >4.5 cm.

Ubranski et al [52] carried out a case-matched study that showed that replacement of the ascending aorta and AV can be performed with similar operative risk, valve-related mortality and late cardiac mortality as isolated AVR. They analysed 100 patients with AV disease and aneurysm (diameter >4.5 cm) of the aorta who underwent AVR and ascending aorta replacement (± complete root) and a matched group of patients undergoing just an AVR. There was no significant difference in the early mortality. 5-year survival seen in the ARR group was 60.7% compared to 86.3% in the AVR group (p = 0.13). At a mean follow-up period of 37 ± 17 months the freedom from cardiac deaths was almost identical in both groups. Similarly, Sundt and colleagues [53] in a retrospective analysis comparing ARR with separate valve and ascending aorta replacement reported no significant difference in early mortality, but showed better survival for the complete root replacement at 5.6 years follow-up (p = 0.04).

Another surgical approach suggested for the management of BAV, associated with a dilated ascending aorta < 5.5 cm, is to carry out AVR with wrapping of the ascending aorta. This method has a low morbidity and mortality rate and was seen to decrease the risk of further dilatation, aneurysm formation and dissection [54,55]. More recently this procedure has been carried out using an external support made to fit the patient's aorta. This was done using digital information from magnetic resonance images to make a replica of the patient's aorta and then computer-aided design to produce the tailored graft [56].

### Suitability of the Ross procedure in BAV

The Ross and Ross-Konno procedure allows the replacement of a stenotic or regurgitant AV in children and young adults with congenital AV disease. Dilatation of the pulmonary autograft root is a common complication following the Ross procedure. The pulmonary and aortic roots share a common embryological origin and it has been proposed that the dilatation of the pulmonary autograft may occur as a result of an intrinsic abnormality within the wall, as seen in the aorta of patients with congenital AV disease [57]. This theory is supported by the findings of de Sa et al [46], that the degenerative changes in the ascending aorta and pulmonary trunk of patients with BAV were more severe than those with TAV. However, this result has been contradicted by Schmid et al [58], who failed to show an association between morphological abnormalities in the dilated aorta and pulmonary artery, in patients with either BAV or TAV. They did, however, confirm the finding of more severe degenerative changes in the aorta associated with BAV. Risk factors for late dilatation of the pulmonary autograft include younger patients, preoperative aortic aneurysm, BAV and those having ARR without support of the annulus and STJ [57,59]. Inclusion techniques have also been described whereby the pulmonary aoutograft is encased in a Dacron tube to prevent dilatation [60].

Hraska et al [61] analysed the mid-term results of 66 children who had undergone the Ross procedure. The mortality rate approached zero in both simple and complex left heart lesions, including infants and neonates. Their main concern was dilatation of the neo-aortic root leading to progression of AR, especially in patients with BAV. Bogers and colleagues analysed 123 patients, 81 with BAV who underwent the Ross procedure at a median follow-up of 5.3 years. Freedom from allograft and autograft intervention was similar in both groups [62].

### Other therapeutic modalities

#### B-blockers

B-blockers decrease the sheer stress of the vessel wall distal to a stenotic lesion. B-blockers are used as long-term therapy in Marfan's syndrome to reduce the rate of pressure change in the aortic root. This is achieved by using their negative inotropic and chronotropic effects to reduce the impulse of the left ventricular ejection and decrease the heart rate. This has been shown to decrease the rate of aortic dilatation and reduce the development of aortic complications [63]. However, no studies have been carried out in BAV patients.

In animal models of AR β-blockers have been shown to reduce the ventricular dilatation and improve remodeling.

### Angiotensin-converting enzyme (ACE) inhibitors and statins

Aortic stenosis and atherosclerosis share several common risk factors: male sex, older age, hypertension, diabetes, smoking and elevated levels of low-density lipoprotein (LDL) cholesterol and lipoprotein(a) [1,7]. These observations have led to the proposal of pharmacological strategies, already used in atherosclerosis, e.g. ACE inhibitors and hydroxymethylglutaryl-coenzyme A reductase inhibitors (statins), which may slow the progression of AS.

ACE has been shown to play an important role in development of atherosclerosis, presumably via its proinflammatory effects. ACE has been found to be present in aortic sclerotic and stenotic valves, but is not found in normal aortic valves, where it may participate in lesion development, as is evidenced by the presence of its enzymatic product, angiotensin II. The observed association between ACE and LDL in both lesions and plasma suggests that LDL may deliver ACE to the lesions [64]. It has previously been shown that ACE inhibitors slow calcium accumulation in aortic valves but a recent study found that ACE inhibitors did not slow the haemodynamic progression rate of AS [65].

Statins are used in the prevention and treatment of atherosclerosis by reducing the levels of LDL cholesterol and lipoprotein(a). Statins have also been shown to slow aortic valve calcium accumulation and a recent study has found that they significantly reduce the haemodynamic progression of both mild-moderate and severe aortic stenosis. This rate of haemodynamic progression was unrelated to cholesterol levels and it is therefore thought that the effects of statins at the valvular level maybe due to their pleiotropic or anti-inflammatory properties rather than by their cholesterol-lowering effects [66]. However, as stated previously, the SALTIRE study group [8] found that treatment with atorvastatin did not halt the progression of calcific aortic stenosis or induce its regression. The anti-inflammatory effects, independent of their lipid-lowering effects, of statins have also been implicated as the mechanism by which they have been shown to suppress the development of experimental abdominal aortic aneurysms (AAA) in normal and hypercholesterolaemic mice. AAAs are associated with atherosclerosis, chronic inflammation and matrix metalloproteinase (MMP)-mediated connective tissue destruction. Statins were shown to preserve the medial elastin and smooth muscle cells and to alter the aortic wall expression of MMPs and their inhibitors [66].

### Calcium antagonists

These have been used in Marfan's syndrome to reduce the rate of dilatation of the aortic root [67]. No studies have yet been carried out in BAV patients but they may have a role in decreasing the rate of post-stenotic dilatation.

### N-terminal B-type natriuretic peptide

B-type natriuretic peptide (BNP) and N-terminal BNP (NtBNP) are neurohormones synthesized and secreted mainly by the ventricular myocardium. An increase in synthesis of NtBNP is associated with wall stress. Natriuretic peptides have been reported to be independent predictors of outcome in congestive heart failure, primary pulmonary hypertension, acute myocardial infarction and pulmonary embolism. Plasma levels of natriuretic peptides are known to be related to disease severity and symptomatic status in AS [68,69] and now preoperative NtBNP has been shown to predict postoperative outcome with regard to survival, symptomatic status and left ventricular function in severe AS [70]. As yet, this has not been measured in aortic disease.

### Synthetic MMP inhibitors (MMPIs)

The MMPs are involved in many physiological functions and, therefore, general inhibition may not be feasible and specific inhibition may be required. The activity of MMPs is kept under tight control at the level of transcription, activation of latent proenzymes and inhibition of proteolytic activity. Modulation of MMP regulation can occur at various biochemical sites. Therapeutic manipulation of the extracellular matrix has been used in other disease processes, including arthritis and malignancy. Possible mechanisms of MMP inhibition include: increasing the levels of the naturally occurring inhibitors (TIMPs), by either exogenous administration of recombinant TIMPs or by increasing their local production; administration of synthetic inhibitors; and decreasing the production of MMPs.

Synthetic inhibitors of MMPs have been investigated in other disease processes, including the use of minocycline in rheumatoid arthritis, and several are currently under investigation for the use in cardiovascular disease [35].

### Conclusion

Post-stenotic aortic dilatation is most commonly seen in patients with a BAV. The degree of this dilatation, however, may not be related to the degree of AS and does not appear to be influenced by the occurrence of AVR. It is likely that the dilatation of the ascending aorta is due, mainly, to intrinsic pathology within the aortic wall rather than the haemodynamic effects of a dysfunctional AV.

There is controversy as to whether the ascending aorta should be replaced at the time of initial AV surgery, if the diameter of the aorta is < 5.5 cm. Most of the evidence suggests that the aorta will continue to dilate at an unknown rate. With improvements of surgical technique and perioperative management resulting in decreased morbidity and mortality following ARR, replacement of the ascending aorta should probably be considered for diameters of 4.5 – 5.5 cm. Other therapeutic strategies being investigated that may reduce the rate of dilation are β-blockers, statins and the new synthetic MMP inhibitors.

#### Competing interests

The author(s) declare that they have no competing interests.
